# Specialty-Specific Entrustable Professional Activities: A Bridge to Internship

**DOI:** 10.7759/cureus.35547

**Published:** 2023-02-27

**Authors:** Karim Hanna, Shanu Gupta, Rebecca Hurst, Bri Anne McKeon, Deborah DeWaay

**Affiliations:** 1 Department of Family Medicine, University of South Florida Morsani College of Medicine, Tampa, USA; 2 Department of Internal Medicine, University of South Florida Morsani College of Medicine, Tampa, USA; 3 Department of Neurology, University of South Florida Morsani College of Medicine, Tampa, USA; 4 Department of Obstetrics and Gynecology, University of South Florida Morsani College of Medicine, Tampa, USA; 5 Department of Medical Education, University of South Florida Morsani College of Medicine, Tampa, USA

**Keywords:** family medicine, obstetrics & gynecology, internal medicine, all neurology, neurology, graduate medical education (gme), gme, ume, education, epa

## Abstract

Background

Undergraduate medical education aims to prepare learners to become capable residents. New interns are expected to perform clinical tasks with distant supervision reliant on having acquired a medical degree. However, there is limited data to discuss what entrustment residency programs grant versus what the medical schools believe they have trained their graduates to perform. At our institution, we sought to foster an alliance between undergraduate medical education (UME) and graduate medical education (GME) toward specialty-specific entrustable professional activities (SSEPAs). These SSEPAs create a bridge to residency and help students structure the final year of medical school while striving for entrustability for day one of residency. This paper describes the SSEPA curriculum development process and student self-assessment of competence.

Methodology

We piloted an SSEPA program with the departments of Family Medicine, Internal Medicine, Neurology, and Obstetrics & Gynecology. Utilizing Kern’s curriculum development framework, each specialty designed a longitudinal curriculum with a post-match capstone course. Students participated in pre-course and post-course self-assessments utilizing the Chen scale for each entrustable professional activity (EPA).

Results

A total of 42 students successfully completed the SSEPA curriculum in these four specialties. Students’ self-assessed competence levels rose from 2.61 to 3.65 in Internal Medicine; 3.23 to 4.12 in Obstetrics and Gynecology; 3.62 to 4.13 in Neurology; and 3.65 to 3.79 in Family Medicine. Students across all specialties noted an increase in confidence from 3.45 to 4.38 in Internal Medicine; 3.3 to 4.6 in Obstetrics and Gynecology; 3.25 to 4.25 in Neurology; and 4.33 to 4.67 in Family Medicine.

Conclusions

A specialty-specific curriculum utilizing a competency-based framework for learners traversing the UME to GME journey in the final year of medical school improves learner confidence in their clinical abilities and may lead to an improved educational handoff between UME and GME.

## Introduction

The undergraduate medical education (UME) system aims to prepare learners to become competent residents [1]. One key step in this process is the acquisition of knowledge, skills, and attitudes needed to matriculate successfully into residency. Trainees value residency preparation courses [2]. However, program directors have expressed that some medical school graduates are not adequately prepared [3-5], and have expressed the utility of the fourth year of medical school as a critical time to develop preparatory skills for the internship [6]. To help bridge this gap the American Association of Medical Colleges (AAMC) released a list of 13 Core Entrustable Professional Activities for Entering Residency (CEPAERs) that a graduating medical student should be able to perform with distant supervision on day one of residency [7]. Evidence from the pilot programs employing the CEPAERs is building to demonstrate the developmental, time-independent nature of these competency-based activities in the workplace [8-10] but tends to target trainees at the level of the clerkship with little literature for pre-clerkship and fourth-year curricula [11].

Upon attempting to implement a pilot program based on the CEPAERs, we discovered two hurdles that had to be overcome. First, recognizing that the UME system is not the system that has to trust the learner on day one of residency. Second, recognizing that it is the graduate medical education (GME) system that will be vulnerable when the learner is performing tasks with distant supervision on day one of residency [12].

What a student had to demonstrate to be considered entrustable varied by specialty at our university. Thus, leaders from our UME/GME programs collaborated to develop specialty-specific entrustable professional activities (SSEPAs). Our goal with SSEPAs was to create a defined curricular structure for the final year of medical school using the entrustable professional activity (EPA) framework for both curricular design and assessment while providing a better bridge to specialty residency for the students. There were two ultimate goals for the SSEPAs articulated by the GME program for the departments. First, the SSEPAs should be highly useful on the first day of residency. Second, the students should perceive a benefit in preparation for the first day of residency. Here, we report our approach to the curriculum development process and preliminary student-level outcomes.

## Materials and methods

Four specialties were chosen to pilot SSEPAs, namely, Family Medicine, Internal Medicine, Neurology, and Obstetrics and Gynecology in the academic year 2019-2020, with a plan for incremental expansion to additional specialties. Specialties were chosen based on departmental capacity to support this project. A faculty representative from each of the four pilot departments was selected one year before the anticipated curricular implementation. A 0.1 faculty time equivalent (FTE) of dedicated academic time was funded by the medical school to an SSEPA lead for curriculum development. This faculty member was charged with establishing an expert panel to identify SSEPAs to expand upon the CEPAERs framework. The department committed to contributing the time of key GME leadership to support the SSEPA lead in choosing the SSEPAs and determining what assessment would be necessary to deem the student entrustable. Subsequently, a curriculum was designed to integrate teaching, learning, and assessment of entrustability within a fourth-year specialty track. The overarching goal for the curriculum was to deliver to the residency programs at our institution interns who could perform SSEPAs with distant supervision where feasible under Accreditation Council for Graduate Medical Education (ACGME) regulations.

Each specialty employed Kern’s framework to develop a curricular blueprint [13]. SSEPAs were identified via an iterative consensus process among the departmental educational leadership that included the Department Chairperson, Residency Program Director, Associate Residency Program Director, Specialty Track Director, Acting Internship Director, Clerkship Director, and Vice Chair of Education. These SSEPAs were mapped to the CEPAERs. However, the goal was not to create SSEPAs for each of the 13 core EPAs but rather to prioritize the EPAs most valuable for each specialty at the start of the internship and reframe these EPAs to specify the specialty context. This approach was chosen to match the resources invested to the needs of the GME program. Entrustment was measured using the Chen scale [14]. Narratives to anchor performance to levels of entrustability were created to accompany each SSEPA and incorporated into our web-based data management system (eValue by MedHub) to track student progress. Each SSEPA leader relayed the EPA material to their department through faculty development meetings and presentations at grand rounds.

Departmental educational leadership, including the third-year clerkship directors and fourth-year acting internship directors, mapped the SSEPAs to the clinical curriculum for feasibility. As an example, it was determined that “gathering an obstetric history” could be evaluated adequately during the fourth-year acting internship.

It was felt that high-risk content and procedures likely could not be evaluated in the clinical setting, as adequate exposure is not guaranteed. Therefore, a two-week course in the spring of the fourth year of medical school was created to assess students in a standardized environment. The curriculum for this course was developed by each EPA lead and varied by specialty. Each included high-yield didactics, case-based discussion, observed structured clinical examinations (OSCEs), and simulation with faculty evaluation, feedback, and remediation. The goal of this two-week course was to assess entrustability and increase the confidence of each student who had matched in the specialty before graduation and matriculation into residency. This curriculum was then presented to the College of Medicine Curriculum Committee for review and approval. This course was planned to proceed in March 2020; however, given the COVID-19 global pandemic, the course content was transitioned to an online format where the content was delivered and assessed virtually.

The authors utilized Kern’s framework to develop a curricular blueprint (Table 1) [13].

**Table 1:** Creation of curricular blueprint using Kern’s framework of curriculum development. UME = undergraduate medical education; GME = graduate medical education; SSEPA = specialty-specific entrustable professional activity; EPA = entrustable professional activity

Problem identification	Targeted needs assessment	Goals and objectives	Educational strategies	Implementation	Evaluation
Readiness for residency; discussion with UME and GME leads; literature review	Gap identification in the skillset of the entering intern; SSEPAs identification in four specialties	Delineation of components and specific learning objectives for each SSEPA	Content delivery through a learning management system; virtual workshops	Final year specialty track curricular alignment; faculty development; longitudinal learner coaching	Level of entrustment defined by behavioral anchors for each EPA using Chen scale; learner survey; faculty survey

A sample blueprint for the creation of the SSEPAs is included (Table 2), and detailed EPAs for each specialty can be found in Appendices.

**Table 2:** Sample SSEPA blueprint. ^1^: Behavioral anchors of the Chen scale. SSEPA = specialty-specific entrustable professional activity; EPA = entrustable professional activity

SSEPA	Subcomponent	Learning objectives	Final EPA assessment^1^
Difficult conversations	Informed consent, e.g., blood transfusion, bedside procedure	1. Identify components of informed consent	1 = Able to observe EPA
			2 = Able to practice EPA with complete direct supervision of an attending
			3 = Able to practice EPA with complete direct supervision of a senior resident
			4 = Able to practice EPA with distant supervision, but an attending/senior resident is immediately available and reviews all findings
			5 = Able to practice EPA with distant supervision, but an attending/senior resident is immediately available and reviews key findings
		2. Communicate specific medical facts to a patient in an understandable way	1 = Able to observe EPA
			2 = Able to practice EPA with complete direct supervision of an attending
			3 = Able to practice EPA with complete direct supervision of a senior resident
			4 = Able to practice EPA with distant supervision, but an attending/senior resident is immediately available and reviews all findings
			5 = Able to practice EPA with distant supervision, but an attending/senior resident is immediately available and reviews key findings
		3. Negotiate a mutually agreeable treatment plan with a patient and confirm the patient’s understanding	1 = Able to observe EPA
			2 = Able to practice EPA with complete direct supervision of an attending
			3 = Able to practice EPA with complete direct supervision of a senior resident
			4 = Able to practice EPA with distant supervision, but an attending/senior resident is immediately available and reviews all findings
			5 = Able to practice EPA with distant supervision, but an attending/senior resident is immediately available and reviews key findings

The initial iteration resulted in a two-week post-match virtual experience with a combination of didactics, small group exercises, and reflective exercises. Many clinical experiences that were designed were abandoned due to restrictions on student exposure due to the COVID-19 pandemic. However, the content creation and delivery satisfied all four specialty leaders. Student feedback was not collected uniformly and thus is not reported here.

In the subsequent iteration (the academic year 2020-2021), the SSEPAs were integrated into the longitudinal specialty tracks where the content was delivered asynchronously utilizing our web-based learning management system, Canvas, and synchronously in-person or virtually. During the Acting Internship and some elective rotations, the students were expected to demonstrate EPAs in situ and receive feedback using the Chen assessment scale as adapted to each EPA. Finally, students attended a two-week post-match capstone experience with a mixture of didactic, simulated, and experiential learning and assessment encounters for each of the SSEPAs.

A pre-course self-assessment was delivered electronically to each student at the start of the final two-week course and a post-course self-assessment was delivered at the end. Students were asked to assess their performance in each SSEPA component utilizing a Chen scale. Additionally, they were asked to rate their confidence in performing these SSEPAs in their residency programs on a five-point agreement Likert scale. Due to the small sample size and heterogeneity of data collected, results are reported as averages only.

As a curricular innovation program evaluation, this study was not required to undergo review by the University of South Florida institutional review board.

## Results

A total of 42 students participated in these four specialties SSEPA curriculum, with 22 in Internal Medicine, 10 in Obstetrics and Gynecology, four in Neurology, and six in Family Medicine (Table 3).

**Table 3:** Retrospective pre-post survey results of student self-assessment and Course Director assessment. *EPA rating key: 1 = Able to observe EPA; 2 = Able to practice EPA with complete direct supervision of an attending; 3 = Able to practice EPA with complete direct supervision of a senior resident; 4 = Able to practice EPA with distant supervision, but an attending/senior resident is immediately available and reviews all findings; 5 = Able to practice EPA with distant supervision, but an attending/senior resident is immediately available and reviews key findings **: Five-point agreement Likert scale: 1 = Strongly disagree; 2 = Disagree; 3 = Neutral; 4 = Agree; 5 = Strongly agree EPA = entrustable professional activity

	Specialty	Internal Medicine (N = 22)		Obstetrics & Gynecology (N = 10)		Neurology (N = 4)		Family Medicine (N = 6)	
		Average student score across all EPAs*	I feel confident to perform these specialty-specific EPAs in my residency program**	Average student score across all EPAs*	I feel confident to perform these specialty-specific EPAs in my residency program**	Average student score across all EPAs*	I feel confident to perform these specialty-specific EPAs in my residency program**	Average student score across all EPAs*	I feel confident to perform these specialty-specific EPAs in my residency program**
Student self-assessment	EPA pre-test average score across the cohort									2.61	3.45	3.23	3.3	3.62	3.25	3.65	4.33
	EPA post-test average score across the cohort	3.65	4.38	4.12	4.6	4.13	4.25	3.79	4.67
Educator assessment of student	EPA average score across the cohort	3.57		3.95		4.6		3.8	

Students in the Internal Medicine cohort on average assessed their performance as a 2.6 on the pre-test and 3.65 on the post-test. On the Chen scale, these correspond to “able to practice EPA with complete direct supervision of an attending” (level 2) and “able to practice EPA with complete direct supervision of a senior resident” (level 3), which is a significant increase in the level of competence. Students in this cohort also noted an increase in confidence from 3.45 in the pre-test to 4.38 in the post-test, moving the needle from “neutral” on the Likert scale to “agree.”

Students in the Obstetrics and Gynecology SSEPA cohort on average scored themselves 3.23 on the pre-test and 4.12 on the post-test. Neurology students similarly scored 3.62 on the pre-test and 4.13 on the post-test. Corresponding to “able to practice EPA with complete direct supervision of a senior resident” (level 3) and “able to practice EPA with distant supervision, but an attending/senior resident is immediately available and reviews all findings” (level 4), respectively, which again represents a significant change in perceived competence. Confidence improved from 3.3 to 4.6 in Obstetrics and Gynecology and 3.25 to 4.25 in Neurology.

Students in the Family Medicine SSEPA cohort on average noted a smaller change in competence, with 3.65 in the pre-test and 3.79 in the post-test. Confidence in this group, however, was notably higher in the pre-test at 4.33 but remained similar in the post-test at 4.67.

The educator assessment of the students on average deemed the students able to practice EPA with complete direct supervision of a senior resident (level 3). Areas, where students did not achieve a level 3 by educator assessment, were limited to the gynecology surgical SSEPA assessing patient positioning, prepping, and draping. Students (N = 5) achieved a level 2, and this was discordant with student self-assessment (3.7).

## Discussion

The final year of the UME curriculum provides educators with an opportunity for the consolidation of knowledge and clinical skills [1]. To create a more concrete bridge to GME, this pilot identified and implemented SSEPAs for final-year medical learners utilizing a framework similar to that described by the CEPAER pilot programs [15].

In contrast to the traditional “boot camps” that develop translational skills through brief simulations and other didactics [2], the SSEPA framework provides the opportunity for a longitudinal curriculum for differentiated final-year medical students. Although boot camps have demonstrated success in three distinct domains, including clinical skills, medical knowledge, and confidence, there is a paucity of literature describing competency-based medical education as a framework for learner outcomes [3,4]. Thus, by implementing a competency-based EPA framework [5], the final year of medical school can better align expectations and transitions to GME. This is possible while providing a shared mental model for education leaders and learners. Considering little data exist to demonstrate improved performance during the internship as a result of boot camps in medical school, this combined framework for learner assessment may provide a more robust platform for data collection and therefore better insights into UME-GME learner gaps [14,16,17].

Our goal was to develop learners who may be entrustable to practice with distant supervision where possible in specialty-specific activities appropriate for a starting intern. Our preliminary results suggest students perceive a gain in competence in specialty-specific skills; however, educator assessments suggest most students remain between levels 3 and 4 on the Chen scale in our SSEPAs. Therefore, more work on the curriculum and assessments are needed to move the students to a level of distant supervision on the Chen scale.

Currently, the SSEPA curriculum concludes in a post-match two-week intensive course, and the final assessments are used formatively for student self-development. In the future, it would be ideal to feed-forward student progress to the program into which they have matriculated for residency as a true process of competency-based medical education. Although not a standard at present, engaging local program leadership in developing and implementing SSEPAs may help engender trust between UME and GME programs to smooth the transition for each learner continuing their medical education journey at the same institution. Ultimately, we intend to develop SSEPAs that promote trust for residency program directors at our institution. We also aim to improve student satisfaction with the final-year clinical experience and to provide a structured specialty-specific experience to students. Because 15-25% of our students continue within our GME programs, there are direct departmental benefits from participation in this project.

Limitations to the generalizability of this curricular innovation are noted. As our SSEPAs were developed with local leadership and implemented in a single institution, they may not be globally applicable. However, we hope to add to the emerging literature on SSEPAs within our respective specialty societies. Additionally, the assessment tools developed are yet to be validated but data from the pilot programs will help inform this gap.

The COVID-19 pandemic affected the implementation of the pilot such that content initially designed for in-person activities was delivered online. Although not ideal for content requiring psychomotor skill development, the virtual platform may indeed be a valuable adjunct tool in the delivery of content for a boot camp [18].

Finally, trust is a belief in the learner’s reliability through measurements of competence, risk, and character. Although this curriculum addresses the first two tenets, the authors note a scarcity of literature addressing character in the language of CEPAERs or SSEPAs, and thus a lack of appropriate assessment of character in this pilot project.

## Conclusions

We present our approach to a specialty-specific curriculum utilizing a competency-based framework with CEPAERs for learners traversing the UME to GME journey. Our virtual pilot at the start of the COVID-19 pandemic helped us utilize a virtual platform for delivering and assessing content. On return to in-person clinical work, our SSEPA pilot thus far has demonstrated a gain in confidence in learners in SSEPAs. We hope to build on this pilot a sustainable structure that delivers entrustable learners to all GME programs locally and nationally in a manner where assessment is feasible and the outcomes are trusted by GME.

## Appendices

EPAs by specialty

**Table 4:** EPAs by specialty. EPA = entrustable professional activity; NIHSS = National Institutes of Health Stroke Scale; IUD = intrauterine device; LP = lumbar puncture

Internal medicine	Obstetrics and gynecology	Neurology	Family medicine
Obtain informed consent (e.g., blood transfusion, bedside procedure)	Gather an obstetric and gynecologic history	Perform a detailed neurologic history and physical	Gather a history and perform a physical examination
Breaking bad news (e.g., medical error, new diagnosis)	Perform a complete breast examination	Perform a history and neurologic examination on a patient with altered mentation/and or coma	Recommend and interpret common diagnostic and screening tests
Multidisciplinary discharge (e.g., team-based communication skills)	Perform a pelvic examination including speculum and bimanual examination in a gynecologic patient	Apply understanding to localize a neurologic problem and generate a differential diagnosis	Provide preventive care that improves wellness, modifies risk factors for illness and injury, and detects illness in early, treatable stages
Overnight urgencies/emergencies (e.g., receiving an overnight call about a patient)	Perform a cervical examination in a pregnant patient including palpation of the cervix and accurate assessment of cervical dilation within 1 cm	Be able to recommend common diagnostic testing and have a basic working knowledge of interpreting the testing ordered (CT head, labs, etc.)	Provide an oral presentation of a clinical encounter
Perform general procedures of a physician: lumbar puncture, thoracentesis, paracentesis, central line placement	Interpret a fetal heart rate tracing and initiate appropriate management	Document a clinical encounter in the patient record with appropriate clinical reasoning	Diagnose and manage chronic medical conditions and multiple co-morbidities
	Perform and interpret a wet mount for common obstetric and gynecologic complaints	Provide an oral presentation of a clinical encounter with reasoning and explanation of results of diagnostic testing and plan of care	Prioritize a differential diagnosis following a clinical encounter
	Provide an oral presentation following a clinical encounter including assessment, differential diagnosis, and plan	Be able to recognize, triage, and perform initial management of neurologic emergencies	Form clinical questions and retrieve evidence to advance patient care
	Perform and receive patient handoffs effectively to transition care	Perform and receive patient handoffs to transition care responsibly	Care for patients and families in multiple settings
	Perform standard maneuvers of a vaginal delivery	Collaborate as a member of an interprofessional team	Evaluate and manage undifferentiated symptoms and complex conditions
	Identify and perform initial evaluation and management for common obstetric emergencies A: obstetric hemorrhage; shoulder dystocia; umbilical cord prolapse	Recognize and perform initial evaluation and management of a stroke patient (NIHSS)	Enter and discuss orders and prescriptions
	Obtain informed consent for basic office and surgical procedures A: IUD insertion; dilation and curettage; cesarean delivery; bilateral tubal ligation	Obtain informed consent for lumbar puncture	Document a clinical encounter in the patient record
	Perform adequate counseling regarding contraceptive options	Perform core procedure of neurology (LP)	Provide first-contact access to care for health issues and medical problems
	Demonstrate basic gynecological surgical principles A: patient positioning; prepping; draping	Identify system failures and contribute to a culture of safety and improvements	Perform general procedures of a physician
	Demonstrate basic surgical skills A: Foley catheter placement; knot tying; simple suturing		Perform common procedures in the outpatient or inpatient setting
